# Spruelike Enteropathy Associated with Olmesartan: An Unusual Case of Severe Diarrhea

**DOI:** 10.1155/2013/618071

**Published:** 2013-03-13

**Authors:** Stephanie E. Dreifuss, Yutaka Tomizawa, Nicholas J. Farber, Jon M. Davison, Adam E. Sohnen

**Affiliations:** ^1^University of Pittsburgh, Graduate School of Medicine, 401 Scaife Hall, 3550 Terrace Street, Pittsburgh, PA 15261, USA; ^2^University of Pittsburgh Medical Center, Department of Internal Medicine, 5230 Centre Avenue, Pittsburgh, PA 15232, USA; ^3^University of Pittsburgh Medical Center, Department of Pathology, 300 Halket Street, Pittsburgh, PA 15213, USA

## Abstract

A 64-year-old male with a history of hypertension presented with worsening diarrhea and 25-pound weight loss over the preceding three months. Prior screening colonoscopy was unremarkable, and the patient failed conservative management. On presentation, the patient had orthostatic hypotension associated with prerenal azotemia for which olmesartan (40 mg/day) was held. Initial workup for chronic diarrhea was essentially unremarkable. Then, EGD was performed with small bowel biopsy, which showed a moderate villous blunting and an intraepithelial lymphocyte infiltration. Celiac disease was excluded by negative conventional serology tests and the absence of clinical response to a gluten-free diet. In the interim, diarrhea became resolving without any other interventions, and clinical response was achieved even with gluten-containing diet. Two months later, he achieved a complete resolution of diarrhea and regained 20-pound weight. Spruelike enteropathy is a clinical entity manifested by chronic diarrhea and intestinal villous atrophy. Spruelike enteropathy associated with olmesartan as a cause of drug-induced diarrhea is rare, and it has been reported only in a case series to date. This case highlighted the importance for clinicians to maintain a high index of suspicion for olmesartan as a precipitant of spruelike enteropathy.

## 1. Introduction

Olmesartan is one of several angiotensin II receptor blocking (ARB) agents. Olmesartan has been shown to have a longer half-life and a greater effect on systolic blood pressure than other ARB agents, making it widely prescribed for the management of hypertension. Diarrhea is a common adverse effect of many medications, and it is one of the common adverse effects of olmesartan. However, in most cases the mechanisms underlying diarrhea remain unclear. Spruelike enteropathy is a clinical entity manifested by chronic diarrhea and intestinal villous atrophy. Spruelike enteropathy as a cause of drug-induced diarrhea has been recognized in posttransplant patients while taking immunosuppressive medications [1–3]. Subsequently, a case series of spruelike enteropathy associated with olmesartan in transplant-naïve patients has recently been reported from a tertiary referral center [4], but no similar reports have been reported to date.

## 2. Case Presentation

**Figure 1:**
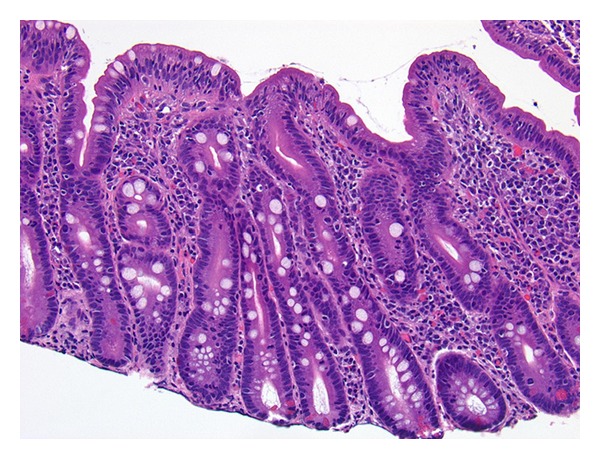
Small intestinal biopsy (×20) showing severely attenuated villi with surface intraepithelial lymphocytosis and lymphoplasmacytic inflammatory infiltrate in the superficial lamina propria.

A 64-year-old male with a past medical history of hypertension presented with worsening diarrhea and 25-pound weight loss over the preceding three months. He reported 5–10 daily episodes of watery, nonbloody diarrhea associated with abdominal bloating. The patient failed conservative management including an opioid-receptor agonist and gluten-free diet as well as a trial of oral antibiotic for possible small bowel bacterial overgrowth. He denied any other symptoms suggestive of local or systemic infections, recent travels or sick contacts, prior exposure to health care environments, or changes in his diet habit or medications within the last few years. Prior screening colonoscopy was unremarkable. On presentation, the patient had orthostatic hypotension for which olmesartan (40 mg/day) was held. Physical examination was unremarkable except for the finding of moderate dehydration. Laboratory data were pertinent for K 3.3 mMol/L, HCO_3_ 19 mMol/L, BUN 44 mg/dL, and Cr 3.1 mg/dL (all the variables were previously documented as normal). Steatorrhea was documented by quantitative measurement of stool fat with microscopic examination revealing >100 stained fat droplets per high power field. Other initial workup including serum thyroid stimulating hormone, stool cultures, stool Clostridium difficile toxin assay, stool ova and parasites, stool osmolality, and stool electrolytes was unrevealing. After recovery from acute prerenal azotemia, a CT scan of abdomen and pelvis was performed, and it was negative for pancreatic abnormality or other suspicious lesions for malignancy. Serum levels of gastrin, vasoactive intestinal poplpeptide, chromogranin A, and urine 5-hydroxyindoleacetic acid (HIAA) level came back to normal limits. A colonoscopy with random colonic biopsies revealed no evidence of microscopic colitis or inflammatory bowel disease. Then, EGD was performed with small bowel biopsy, which showed moderate villous blunting and intraepithelial lymphocyte infiltration without subepithelial collagen depositions (Figure 1). However, both IgA tissue transglutaminase (TTG) antibody and IgA tissue endomysial antibody were negative. HLA-DQ2 and DQ8 were not present. Celiac disease was excluded by negative conventional serology tests and the absence of clinical response to a gluten-free diet. In the interim, the frequency and amount of stool became decreasing, and the patient gradually became tolerating oral intake. No medication was needed to control diarrhea after clinical response was achieved even with gluten-containing diet. He was discharged to home with suspension of olmesartan. At a two-week followup, he reported 1-2 formed stools a day without abdominal symptoms. Two months later, he achieved complete resolution of diarrhea and regained 20-pound weight. He has been well and continued under observation.

## 3. Discussion

We described a case of severe spruelike enteropathy which achieved a remarkable clinical improvement with suspension of olmesartan. Small bowel biopsy confirmed moderately severe spruelike manifestations including villous blunting and intraepithelial lymphocyte infiltration. Celiac disease was excluded by negative conventional serology tests, the absence of HLA class II heterodimer HLA-DQ2 or HLA-DQ8, and the absence of clinical response to a gluten-free diet. Virtually, all patients with celiac disease have the celiac disease-associated alleles, the DQ2 or DQ8 molecule [5]. Thus, the presence of those alleles provides a sensitivity close to 100% for celiac disease and a very high negative predictive value for the disease [6]. Biopsy did not show subepithelial collagen depositions, increased crypt apoptosis, or crypt predominant lymphocytosis. Other less common enteropathies were excluded.

This case highlighted very similar clinicopathological manifestations presented in the previous case series of spruelike enteropathy associated with olmesartan [4]. The study by Rubio-Tapia et al. described a group of 22 patients (13 women, median age of 69.5 years) who presented with severe diarrhea and weight loss with a median of 18 kg loss. Most patients had been taking 40 mg of olmesartan daily for several months or years (average of 3.1 years) before developing symptoms. Clinical response was observed in all the patients after suspension of olmesartan. In all patients, results of IgA TTG antibody were negative, a gluten-free diet was not helpful, and baseline intestinal biopsies demonstrated villous atrophy with mucosal inflammation. Followup intestinal biopsies were performed in 18 patients (82%), and histologic recovery of the duodenum was documented in 17 patients. We acknowledge that this case lacks information of histologic recovery after discontinuation of olmesartan. However, given the achievement of clinical remission, it is very unlikely that the presence of villous blunting or mucosal inflammation persisted. Deliberate rechallenge test with olmesartan was not indicated because of the life-threatening nature of the syndrome. Resolution of the symptoms in the absence of clinicopathological evidence of other diseases associated with enteropathy suggests that the association is not likely to be a chance as the previous report by Rubio-Tapia et al. mentioned as well. The mechanisms underlying spruelike enteropathy associated with olmesartan remain unclear. Given the time between the initiation of olmesartan and the development of symptoms, it is unlikely that clinical picture is the result of a type I hypersensitivity response. One proposed mechanism is that the enteropathy is due to a cell-mediated immune response that results in damage to the small intestinal brush border. Angiotensin II induces gene expression of transforming growth factor- (TGF-) beta. The upregulation of TGF-beta levels is involved in damage to various organs, and the gene expressions are induced by angiotensin II [7]. In the gastrointestinal tract, TGF-beta plays a central role in the intestinal epithelial cells and the mucosal immune system to maintain a normal balance between proinflammatory and antiinflammatory factors [8]. In conclusion, we report a very unique case describing an association of severe form of spruelike enteropathy and olmesartan. Although olmesartan has not been proved for the causality of spruelike enteropathy, our case supports the findings in the recently published first case series. Further investigation is warranted to elucidate the specific mechanism of olmesartan-associated enteropathy and to determine whether other drugs in the ARB class can result in a similar adverse reaction. Until these questions are answered, it is important for clinicians to maintain a high index of suspicion for olmesartan and potentially other ARBs as precipitants of spruelike enteropathy.
